# Agreement between a web collaborative dataset and an administrative dataset to assess the retail food environment in Mexico

**DOI:** 10.1186/s12889-024-18410-3

**Published:** 2024-04-01

**Authors:** Yenisei Ramírez-Toscano, Daniel Skaba, Vanderlei Pascoal de Matos, Carolina Pérez-Ferrer, Tonatiuh Barrientos-Gutiérrez, Nancy López-Olmedo, Maria de Fátima Pina

**Affiliations:** 1 Center for Population Health Research, National Institute of Public Health, Avenida Universidad 655, Santa María Ahuacatitlán, Cuernavaca, Morelos CP, 62100 Mexico; 2 Instituto de Comunicação E Informação Científica E Tecnológica Em Saúde / Fundação Oswaldo Cruz - ICICT/FIOCRUZ, Rio de Janeiro, Brazil; 3 Instituto de Investigação E Inovação Em Saúde Universidade Do Porto, Porto, Portugal

**Keywords:** Retail food environment, Food outlets, Agreement, Secondary data, Web data

## Abstract

**Background:**

Latin American countries are often limited in the availability of food outlet data. There is a need to use online search engines that allow the identification of food outlets and assess their agreement with field observations. We aimed to assess the agreement in the density of food outlets provided by a web collaborative data (Google) against the density obtained from an administrative registry. We also determined whether the agreement differed by type of food outlet and by area-level socioeconomic deprivation.

**Methods:**

In this cross-sectional study, we analyzed 1,693 census tracts from the municipalities of Hermosillo, Leon, Oaxaca de Juarez, and Tlalpan. The Google service was used to develop a tool for the automatic acquisition of food outlet data. To assess agreement, we compared food outlet densities obtained with Google against those registered in the National Statistical Directory of Economic Units (DENUE). Continuous densities were assessed using Bland–Altman plots and concordance correlation coefficient (CCC), while agreement across tertiles of density was estimated using weighted kappa.

**Results:**

The CCC indicated a strong correlation between Google and DENUE in the overall sample (0.75); by food outlet, most of the correlations were from negligible (0.08) to moderate (0.58). The CCC showed a weaker correlation as deprivation increased. Weighted kappa indicated substantial agreement between Google and DENUE across all census tracts (0.64). By type of food outlet, the weighted kappa showed substantial agreement for restaurants (0.69) and specialty food stores (0.68); the agreement was moderate for convenience stores/small food retail stores (0.49) and fair for candy/ice cream stores (0.30). Weighted kappa indicated substantial agreement in low-deprivation areas (0.63); in very high-deprivation areas, the agreement was moderate (0.42).

**Conclusions:**

Google could be useful in assessing fixed food outlet densities as a categorical indicator, especially for some establishments, like specialty food stores and restaurants. The data could also be informative of the availability of fixed food outlets, particularly in less deprived areas.

**Supplementary Information:**

The online version contains supplementary material available at 10.1186/s12889-024-18410-3.

## Background

The retail food environment is a key driver for dietary change, as it facilitates the consumption of healthy or unhealthy foods at the population level [1]. Recent studies have shown an association between the number of food stores and food intake behaviors [2, 3] and diet-related outcomes [4, 5], suggesting that food store availability could represent a venue for interventions to increase the healthfulness of diets. An important challenge to advancing the study of the retail food environment is the assessment of food store availability, especially in low- and middle- income countries.

Primary data collection is the gold standard to identify the number of food stores in an area, a proxy for food store availability [6, 7]. However, this approach is time-consuming and expensive [8]. Secondary data, such as food license registries or private business datasets have been widely used [7], yet they are often unavailable in some areas or countries, especially where the proportion of informal business is high. In recent years, the possibility of mining crowdsourced data has become an opportunity to assess food store availability. Geographic Information Systems (GIS) online search-engines allow for identification of sites by pointing to a location on a map, inputting an individual address, or by importing addresses from a database [9, 10]. Google provides detailed information on a wide variety of food stores that is updated on an everyday basis, obtained by their own field survey, partner organizations, and end users [11, 12]. This represents a potential source of information for low- and middle-income countries without administrative records, yet few efforts have been made to assess the potential validity of Google data to evaluate food store counts in their specific economic context.

The validity of GIS-based tools, such as Google Maps or Google Street View, has been evaluated in high-income countries against primary data collection (field audits or street audits) [6, 13, 14]. For instance, one study from Norway found a moderate to almost perfect validity [13], and another study from Spain observed a high level of agreement to characterize food stores [14]. In Latin America, a recent study in Brazil used a tool to mine the community food retail through Google Earth (GE) and found moderate to excellent validity compared to ground-truth data [15]. This study showed promise, yet, it was restricted to two urban territories in Brazil as it would require considerable financing to conduct a nationally representative validation. Further assessments in other Latin American countries and larger areas are needed to inform those countries without primary data on food availability about the potential validity of GIS-based tools considering the economic and social context of Latin America. In particular, validation or agreement studies need to consider differences across territories in socioeconomic deprivation, considering that crowdsourced data requires access to smartphones and internet infrastructure, as well as community participation to be up to date and complete [16]. This is important since people in vulnerable areas probably collaborate less with Google, meaning they do not upload data about the establishments as much as people in more affluent areas.

Taking advantage of the rich food outlet publicly available administrative data in Mexico which represents a reliable source of information with regular ground-truthing by government officials, we aimed to assess the agreement in the density of food outlets provided by Google against the density obtained from the National Institute of Statistics and Geography of Mexico during economic censuses. We also determined whether the agreement differed by type of food outlet and by area-level socioeconomic deprivation. We hypothesized that the agreement of food retail data collected from Google Maps would be comparable to the agreement of food retail data from an administrative dataset overall, but that the agreement would be better in less deprived areas than in more deprived areas.

## Methods

### Study setting and sample

To maximize the variability of living conditions that could affect the quality of data in Google Maps we selected four municipalities from Mexico, each from one region of the country: Hermosillo from the North, Leon from the Center, Tlalpan from Mexico City, and Oaxaca de Juarez from the South. Hermosillo, located in the state of Sonora, has an estimated population of 936,263 inhabitants and covers an area of 16,955 km^2^. Leon, in Guanajuato, covers an area of 1,221 km^2^ and an estimated population of 1,721,215. Tlalpan is in Mexico City and has a population of 699,928 living in an area of 314 km^2^. The municipality of Oaxaca de Juarez is in the state of Oaxaca, with an estimated population of 270,955 and an area of 89 km^2^. The estimated population size and land area were obtained from the 2020 National Census [17] and the 2019 Geostatistical framework, respectively [18]. Also, we selected these municipalities to have variability in economic conditions, internet infrastructure and use of cell phones. The Gross Domestic Product (GDP) in 2020 from Mexico City was the highest (3,464,828 million MXN) followed by Guanajuato (949,404 million MXN), Sonora (784,273 million MXN), and Oaxaca de Juarez (352,163 million MXN) [19]. The highest proportion of internet users was found in Mexico City (84.4%), followed by Sonora (82.9%), Guanajuato (67.3%), and Oaxaca de Juarez (55.0%). Overall, 96.0% of internet users connected through a smartphone [20].

The unit of analysis consisted on all urban census tracts (urban Basic Geostatistical Areas-AGEB in Mexico) in the four municipalities (*n* = 1,721). An urban census tract is a geographical area with socioeconomically homogeneous characteristics occupied by 25 to 50 blocks delimited by streets, avenues, sidewalks, or any other feature of easy identification [21]. Since DENUE but not Google has the capacity to identify each food outlet inside central markets, we excluded those census tracks where it is more likely that a very high density of food outlets in DENUE reflects the presence of central markets. To do so, we used the 99th percentile of the distribution of food outlet density as the cut-off point, for a final sample of 1,693 census tracts. The median area per census tract in our sample was 0.2 km^2^, while the median population was 1,588 inhabitants, being lowest for Hermosillo (857 inhabitants) and highest for Tlalpan (3,015 inhabitants). A summary of characteristics of the four municipalities is available in Supplementary Table 1.

### Administrative dataset: food outlet’s official data

Food outlets were obtained from the National Statistical Directory of Economic Units (*DENUE*). DENUE is conducted by the National Institute of Statistics and Geography of Mexico (*INEGI*) and contains information on the principal economic activity and location of fixed economic units that carry out activities related to manufacturing, commerce, and services [22]. DENUE is an administrative government list (secondary data) based on the National Economic Censuses, the backbone of the National Economic Information Subsystem, representing a direct source of information with regular ground-truthing (every five years) by government officials [23]. The first version of the DENUE was published in July 2010 with information collected by the 2009 National Economic Censuses on active economic units in the national territory. The updating strategies include yearly economic surveys and fieldwork operations to update large businesses and verify the information of the economic units. We used the 2022 DENUE data for the present study, which is based on information from 2021.

We identified and classified each food outlet using the North American Industrial Classification System (NAICS). We included the following food outlets in our analyses categorized as follows: (1) Specialty food stores (bakery and pastry shops, meat markets, fruit and vegetable stores, health food stores), (2) Restaurants (restaurants, bars, coffee shops, fast food restaurants), (3) Candy and ice cream stores, (4) Supermarkets, and (5) Convenience stores/small food retail stores (*tiendas de abarrote*s in Mexico). The definitions, NAICS codes, and examples of the food store categories are available in Supplementary Table 2.

### Google Maps data acquisition

Google Maps provides detailed information on a wide variety of food outlets, mainly fixed obtained by data collected from their own field survey using street view vehicles, but also from thousands of partner organizations around the World (government and non-government agencies), businesses through the use of the Google My Business tool (registering their business and provide up-to-date information) and end users, who provide additional details to businesses like reviews, besides pointing errors to be checked by Google, since users cannot edit the maps [11]. We developed and implemented an algorithm to automatically acquire data from the Google Maps platform. The process is done through the Google Application Programming Interface (Google API), using the API Places and, having as inputs a location (pair of coordinates, name, or address), a search distance, and a type of point of interest. Points of interest in the Google API are each registry in Google´s database classified according to the existing 139 different types in Google. For each search, Google returns a maximum of 60 points of interest, each of them with a unique identification code, geographic coordinates, name of the point of interest, address and classified from 1 up to 8 Google types. From the 139 existing Google types, we selected the 11 types related to food outlets to our searches, specifically: bakery, bar, café (coffee shop), convenience_ store, liquor_store, meal_delivery, meal_takeaway, restaurant, supermarket, grocery_or_supermarket and food store. The acquisition of data was conducted between April and June 2021. Although Google does not provide the date when each POI is added to their database, we can confirm that each food establishment was listed as active in Google at the time of our search.

The algorithm made an initial search in a buffer around the geometric center of a rectangle that encompasses the entire area of study, setting the search for one of the food outlet types and the radius distance to half of the diagonal of the rectangle. If the search returned less than 60 points of interest, this meant all the outlets of the selected type were detected in that area, and the algorithm stopped. If the search returned 60 points of interest (the maximum that Google returns in each search) then the initial rectangle was divided into four equal sub-areas, and the search was repeated in each of these sub-areas This process was done recursively until the search returned less than 60 establishments in each portion. The process was then repeated for each of the 11 selected food outlet types. In the end, duplicate establishments, identified by the unique code, and those located outside the limits of the study area were excluded. The searches were made via URL, and we developed a script in R language to automate the process.

In order to make a classification of the food outlets that could be comparable with the DENUE dataset, we developed an inventory of terminology with 177 words, for food outlet types in Mexico in consensus with local researchers. The list of words for the food outlets is available in Supplementary Table 3. We grouped the points of interest by doing a text search of the 177 points of interest words on their names, through scripts in MySQL; for instance, if the word “restaurante” was in the name of the establishment, then it was classified as restaurant. Finally, we geocoded each point of interest, attributing the code of the census tract where the point of interest is located, by overlaying the geographic coordinates of the point of interest with the Census tract map, using QGIS software. Each Census tract has the deprivation level attribute.

### Socioeconomic deprivation

Census tracts were classified by socioeconomic deprivation using the 2020 marginality index [24]. The National Population Council developed this index for each census tract based on the General Population and Housing Census data. The marginality index is a composite of area-level socioeconomic deprivation that includes nine variables across four dimensions: access to public services, access to education, and economic and employment conditions. The index was divided into quintiles: very low, low, medium, high, and very high deprivation [24]. The stratified agreement analysis by socioeconomic deprivation has a different sample size, as some census tracts do not have information (*n* = 241), therefore for this analysis we used 1,452 census tracts. Half of the census tracts were classified in very low and low socioeconomic deprivation levels. Most of the census tracts from Hermosillo and Tlalpan were in the very low and low socioeconomic deprivation categories. In contrast, most of Leon and Oaxaca de Juarez’s census tracts were in medium and low socioeconomic deprivation levels (Supplementary Table 1).

### Statistical analysis

First, we described the means and medians with measures of dispersion of the density of food outlets from both instruments, Google data and DENUE. We then compared the density of food outlets (counts/area of the census tract in km^2^) between the DENUE database and the Google data, within each census tract, by municipality, type of food outlet, and socioeconomic deprivation using the Wilcoxon signed-rank test. We classified the food outlets into five categories: (1) Specialty food stores, (2) Restaurants, (3) Candy and ice cream stores, (4) Supermarkets, and (5) Convenience stores/small food retail stores.

We evaluated the agreement of Google data with the administrative data (DENUE) by using Bland–Altman plots, a method to describe the agreement between two quantitative measurements [25]. The Bland–Altman plot is a scatter plot in which the difference between the paired measurements (A-B) is plotted against their mean value ([A + B]/2), estimating the mean level of agreement and 95% limits of agreement [26]. We estimated the agreement between Google data and DENUE in the overall sample and by socioeconomic deprivation and food outlet type. As indicated by Bland & Altman [27], we checked the assumption of normality by drawing a histogram of the differences between Google and DENUE data. Normality is assumed if the distribution of the differences is not skewed or has very long tails [27]. The result suggests a roughly normal distribution (see Supplementary Fig. 1).

As we cannot define a priori the limits of maximum acceptable differences between Google and DENUE, we used as a complementary approach a scaled index, the concordance correlation coefficient (CCC) [28]. We estimated the CCC in the overall sample and by socioeconomic deprivation and food outlet type. It has been suggested that the CCC should be interpreted close to other correlation coefficients similar to the Pearson correlation coefficient [29]. There have been several cutoff points to interpret the correlation coefficients; we followed a general guideline as follows: 0.00–0.10 negligible, 0.10–0.39 weak, 0.40–0.69 moderate, 0.70–0.89 strong, and 0.90–1.00 very strong [30].

To assess the categorical agreement between Google data and DENUE we divided the food outlet density on each dataset into tertiles and calculated weighted Cohen’s kappa coefficients to assess agreement. Kappa coefficients were calculated for the overall sample, by socioeconomic deprivation and by food outlet type. We followed Landis and Koch interpretation guidelines for kappa coefficients, as follows: < 0.0 poor, 0.00–0.20 slight, 0.21–0.40 fair, 0.41–0.60 moderate, 0.61–0.80 substantial, and 0.81–1.00 almost perfect [31].

Statistical analyses were performed in Stata 18 (StataCorp, Stata Statistical Software, Release 18, 2023).

## Results

Table 1 shows the mean and median density of food outlets by instrument (Google data and DENUE). The mean density of food outlets by DENUE was 82.9 (SD: 93.0), while in Goggle data was 60.3 (SD: 74.4). The median density of food outlets by DENUE was 56.0 (p25: 7.4, p75: 117.7), while in Google data was 33.8 (p25: 0.0, p75: 92.0). Google data underestimated the density of food outlets in almost all the municipalities, type of food outlets, and levels of deprivation, except for the municipality of Tlalpan, the specialty food store type, the supermarkets, and the very low deprivation category.

**Table 1:** Density of food outlets of Google data and DENUE in census tracts in Mexico, 2021

	**DENUE**	**Google data**		**DENUE**	**Google data**	
Density of food outlets (counts/km^2^)	**Mean (SD)**	**Mean (SD)**	***p*-value**^**a**^	**Median (p25,p75)**	**Median (p25,p75)**	***p*-value**^**b**^
**Overall**	82.9 (93.0)	60.3 (74.4)	< 0.001	56.0 (7.4, 117.7)	33.8 (0.0, 92.0)	< 0.001
**Municipality, State**						
**Hermosillo, Sonora**	41.1 (43.4)	35.2 (42.2)	< 0.001	34.5 (0.0, 66.9)	21.2 (0.0, 56.3)	0.004
**Leon, Guanajuato**	100.4 (106.1)	71.9 (84.9)	< 0.001	68.8 (6.6, 166.9)	38.1 (0.0, 121.9)	< 0.001
**Tlalpan, Mexico City**	120.1 (102.4)	108.8 (89.5)	0.006	96.9 (36.0, 184.9)	104.0 (31.6, 158.6)	< 0.001
**Oaxaca de Juarez, Oaxaca**	140.2 (101.4)	52.5 (67.8)	< 0.001	120.6 (70.1, 190.4)	27.4 (9.2, 67.8)	< 0.001
**Type of food outlets**						
**Specialty food stores**	13.7 (24.3)	26.3 (42.0)	< 0.001	3.0 (0.0, 16.4)	7.9 (0.0, 33.7)	< 0.001
**Restaurants**	30.6 (43.2)	18.1 (24.9)	< 0.001	13.1 (0.0, 45.1)	7.5 (0.0, 29.2)	< 0.001
**Candy and ice cream stores**	4.1 (9.6)	0.8 (2.9)	< 0.001	0.0 (0.0, 4.7)	0.0 (0.0, 0.0)	< 0.001
**Supermarkets**	0.6 (2.6)	0.9 (3.2)	< 0.001	0.0 (0.0, 0.0)	0.0 (0.0, 0.0)	< 0.001
**Convenience stores/small food retail stores**	33.9 (37.6)	11.1 (15.7)	< 0.001	23.0 (0.0, 53.1)	4.8 (0.0, 17.9)	< 0.001
**Socioeconomic deprivation**^c^						
**Very low**	52.8 (60.3)	60.3 (66.6)	< 0.001	37.6 (6.3, 75.2)	41.5 (7.4, 88.5)	< 0.001
**Low**	123.5 (96.3)	100.5 (83.2)	< 0.001	98.1 (52.8, 176.9)	82.4 (35.6, 144.7)	< 0.001
**Medium**	148.9 (113.2)	85.9 (82.8)	< 0.001	125.9 (54.8, 226.5)	55.6 (17.9, 138.8)	< 0.001
**High**	96.4 (86.9)	37.4 (46.5)	< 0.001	77.8 (34.1, 119.1)	21.8 (2.9, 50.2)	< 0.001
**Very high**	42.6 (45.7)	9.8 (16.7)	< 0.001	32.6 (0.0, 65.5)	0.0 (0.0, 15.9)	< 0.001

*DENUE* National Statistical Directory of Economic Units, *SD* Standard Deviation, *p25* 25th percentile, *p75* 75th percentile

^a^Significance (*p* < 0.05) by paired t-test

^b^Significance (*p* < 0.05) by Wilcoxon signed-rank test

^c^Different sample size (*n* = 1452)

**Fig. 1:**
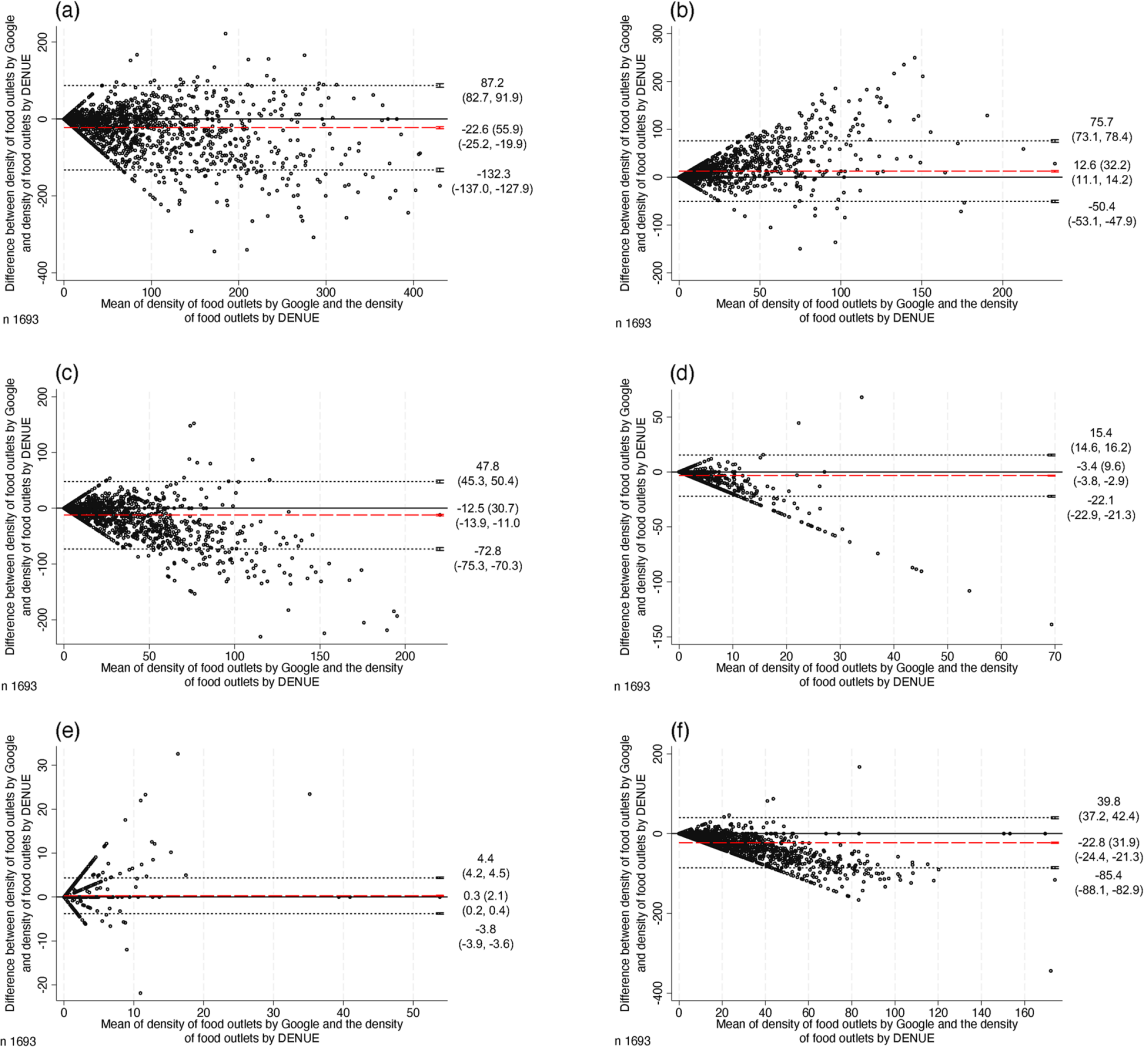
Bland–Altman plots for density of food outlets between Google and DENUE: overall and by food outlets, 2021. (**a**) Overall, (**b**) Specialty food stores, (**c**) Restaurants, (**d**) Candy and ice cream stores, (**e**) Supermarkets, (**f**) Convenience stores/small food retail stores. Dots represent the difference between the density of food outlets by Google and the density of food outlets by DENUE against their mean value in each census tract. Dotted lines represent 95% limits of agreement with their respective 95% confidence interval. Red dashed lines represent the mean difference with their respective 95% confidence interval

Table 2 presents the concordance correlation coefficients, and Fig. 1 the corresponding Bland–Altman plots, overall and by food outlets. In the overall sample, the CCC was 0.75, meaning a strong correlation; the mean difference between Google and DENUE was -22.6. Most of the correlations were from negligible to moderate by type of food outlet. Candy and ice cream stores had the lowest CCC (0.08; mean difference: -3.4), indicating a negligible correlation, while supermarkets had the highest, indicating a strong correlation (CCC: 0.74; mean difference: 0.3).

**Table 2:** Concordance correlation coefficient between Google data and DENUE in census tracts in Mexico, 2021

Density of food outlets (counts/km^2^)	Concordance correlation coefficient (95% CI) between Google and DENUE
**Overall**	0.75 (0.73, 0.77)
**Type of food outlets**	
**Specialty food stores**	0.52 (0.50, 0.55)
**Restaurants**	0.58 (0.56, 0.61)
**Candy and ice cream stores**	0.08 (0.06, 0.10)
**Supermarkets**	0.74 (0.72, 0.76)
**Convenience stores/small food retail stores**	0.29 (0.27, 0.32)
**Socioeconomic deprivation**^a^	
**Very low**	0.82 (0.79, 0.85)
**Low**	0.79 (0.75, 0.82)
**Medium**	0.65 (0.59, 0.70)
**High**	0.49 (0.41, 0.56)
**Very high**	0.27 (0.20, 0.34)

*DENUE* National Statistical Directory of Economic Units, *95% CI* 95% Confidence interval

^a^Different sample size (*n* = 1452)

**Fig. 2:**
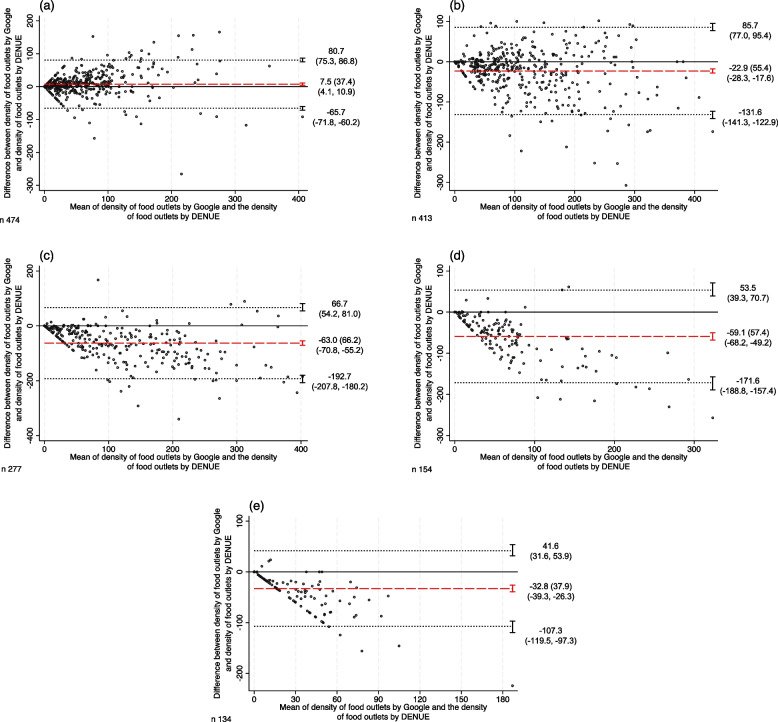
Bland–Altman plots for density of food outlets between Google and DENUE: by socioeconomic deprivation, 2021. (**a**) Very low deprivation, (**b**) Low deprivation, (**c**) Medium deprivation, (**d**) High deprivation, (**e**) Very high deprivation. Dots represent the difference between the density of food outlets by Google and the density of food outlets by DENUE against their mean value in each census tract. Dotted lines represent 95% limits of agreement with their respective 95% confidence interval. Red dashed lines represent the mean difference with their respective 95% confidence interval

Table 2 presents the concordance correlation coefficients, and Fig. 2 the corresponding Bland–Altman plots by socioeconomic deprivation. The CCC showed a weaker correlation between Google and DENUE as deprivation increased. The very low and low deprivation categories showed a strong correlation (CCC: 0.82 and 0.79; mean difference: 7.5 and -22.9, respectively), while the very high deprivation showed a weak correlation (CCC: 0.27; mean difference: -32.8).

Table 3 shows the cross-classification by tertiles of the density of food outlets and weighted kappa between Google data and DENUE. The density of food outlets between Google data and DENUE was categorized in the same tertile (correctly classified) in 70% of census tracts. Weighted kappa indicated substantial agreement when considering all census tracts (0.64). When we analyzed by municipality, the weighted kappa indicated substantial agreement in Leon (0.70), Tlalpan (0.68), and Hermosillo (0.60), while the weighted kappa for Oaxaca de Juarez indicated moderate agreement (0.54).

**Table 3:** Cross classification by tertiles of food outlets between Google data and DENUE, 2021

Density of food outlets (counts/km^2^)	n	Correctly classified (%)^a^	Misclassified (Google data underestimates) (%)^b^	Misclassified (Google data overestimates) (%)^c^	Weighted kappa (SE)
**Total census tracts**	1693	70.1	14.4	15.5	0.64 (0.02)
**Hermosillo, Sonora**	662	67.5	16.2	16.3	0.60 (0.03)
**Leon, Guanajuato**	687	74.5	12.2	13.2	0.70 (0.03)
**Tlalpan, Mexico City**	203	71.4	14.3	14.3	0.68 (0.06)
**Oaxaca de Juarez, Oaxaca**	141	61.7	19.1	19.1	0.54 (0.07)

*DENUE* National Statistical Directory of Economic Units, *SE* Standard Error

^a^Correctly classified = % of census tracts with density of food outlets of DENUE and Google data in the same tertile

^b^Misclassified (Google data underestimates) = % of census tracts adjacently or extreme opposite tertile classified when Google data underestimates

^c^Misclassified (Google data overestimates) = % of census tracts adjacently or extreme opposite tertile classified when Google data overestimates

Table 4 shows agreement in tertiles of the density of food outlets and the weighted kappa between Google data and DENUE by food outlets. A total of 89.1%, 74.7%, 74.4%, 65.7%, and 60.6% of supermarkets, specialty food stores, restaurants, candy/ice cream stores, and convenience stores/small food retail stores, respectively, were categorized in the same tertile by Google data and DENUE in the census tracts under study. The highest weighted kappa was observed for restaurants (0.69) and specialty food stores (0.68), indicating substantial agreement, while the lowest were for convenience stores/small food retail stores (0.49), indicating moderate agreement, and for candy and ice cream stores (0.30), indicating fair agreement.

**Table 4:** Cross classification by tertiles of food outlets between Google data and DENUE: by food outlets, 2021

Density of food outlets (counts/km^2^)	n	Correctly classified (%)^a^	Misclassified (Google data underestimates) (%)^b^	Misclassified (Google data overestimates) (%)^c^	Weighted kappa (SE)
**Specialty food stores**	1693	74.7	10	15.2	0.68 (0.02)
**Restaurants**	1693	74.4	14.8	10.8	0.69 (0.02)
**Candy and ice cream stores**	1693	65.7	30.1	4.1	0.30 (0.02)
**Supermarkets**	1693	89.1	3.5	7.4	0.62 (0.02)
**Convenience stores/small food retail stores**	1693	60.6	20.1	19.3	0.49 (0.02)

*DENUE* National Statistical Directory of Economic Units, *SE* Standard Error

^a^Correctly classified = % of census tracts with density of food outlets of DENUE and Google data in the same tertile

^b^Misclassified (Google data underestimates) = % of census tracts adjacently or extreme opposite tertile classified when Google data underestimates

^c^Misclassified (Google data overestimates) = % of census tracts adjacently or extreme opposite tertile classified when Google data overestimates

Table 5 presents the cross-classification by tertiles of the density of food outlets and weighted kappa between Google data and DENUE by socioeconomic deprivation. Results observed by socioeconomic deprivation were from 59.1% in the high deprivation category to 76.5% in the low deprivation category correctly classified. Although there was not a pattern of agreement across categories of socioeconomic deprivation, we observed a moderate agreement in very high deprivation areas (0.42), with better agreement in low deprivation areas (0.63). Results observed by municipality indicate in general that the agreement is better in the lowest deprivation areas, for instance Hermosillo showed a substantial agreement in the very low deprivation areas (0.66), Leon showed a substantial agreement in the medium deprivation areas (0.67), Tlalpan showed an almost perfect agreement in the medium deprivation areas (0.87), and Oaxaca de Juarez showed a moderate agreement in the very low deprivation areas (0.55).

**Table 5:** Cross classification by tertiles of food outlets between Google data and DENUE: by socioeconomic deprivation and municipality, 2021

Density of food outlets (counts/km^2^)	n	Correctly classified (%)^a^	Misclassified (Google data underestimates) (%)^b^	Misclassified (Google data overestimates) (%)^c^	Weighted kappa (SE)
**Total census tracts**					
Socioeconomic deprivation					
Very low	474	60.8	4.4	34.8	0.52 (0.03)
Low	413	76.5	9.7	13.8	0.63 (0.04)
Medium	277	69.3	24.2	6.5	0.55 (0.05)
High	154	59.1	36.4	4.5	0.46 (0.05)
Very high	134	65.7	32.1	2.2	0.42 (0.06)
**Hermosillo, Sonora**					
Socioeconomic deprivation					
Very low	276	73.2	6.2	20.7	0.66 (0.05)
Low	142	67.6	19.7	12.7	0.49 (0.07)
Medium	41	56.1	34.1	9.8	0.29 (0.11)
High	25	28.0	68.0	4.0	0.18 (0.10)
Very high	46	50.0	37.0	13.0	0.24 (0.11)
**Leon, Guanajuato**					
Socioeconomic deprivation					
Very low	128	60.2	2.3	37.5	0.50 (0.06)
Low	144	75.7	10.4	13.9	0.64 (0.07)
Medium	165	79.4	13.9	6.7	0.67 (0.06)
High	85	75.3	17.6	7.1	0.64 (0.08)
Very high	70	70.0	28.6	1.4	0.45 (0.10)
**Tlalpan, Mexico City**					
Socioeconomic deprivation					
Very low	59	67.8	3.4	28.8	0.63 (0.10)
Low	86	72.1	15.1	12.8	0.60 (0.09)
Medium	27	88.9	11.1	0.0	0.87 (0.16)
High	20	40.0	55.0	5.0	0.12 (0.13)
Very high^d^	-	-	-	-	-
**Oaxaca de Juarez, Oaxaca**					
Socioeconomic deprivation					
Very low	11	63.6	9.1	27.3	0.55 (0.23)
Low	41	70.7	4.9	24.4	0.55 (0.12)
Medium	44	43.2	29.5	27.3	0.20 (0.11)
High	24	60.5	37.5	0.0	0.47 (0.14)
Very high	12	75.0	16.7	8.3	0.40 (0.28)

Sample size: Total census tracts (*n* = 1452); Hermosillo, Sonora (*n* = 530); Leon, Guanajuato (*n* = 592); Tlalpan, Mexico City (*n* = 192); Oaxaca de Juarez, Oaxaca (*n* = 132)

*DENUE* National Statistical Directory of Economic Units, *SE* Standard Error

^a^Correctly classified = % of census tracts with density of food outlets of DENUE and Google data in the same tertile

^b^Misclassified (Google data underestimates) = % of census tracts adjacently or extreme opposite tertile classified when Google data underestimates

^c^Misclassified (Google data overestimates) = % of census tracts adjacently or extreme opposite tertile classified when Google data overestimates

^d^ Too few sample size (*n* = 6) to rating categories

## Discussion

We aimed to assess the agreement of Google food outlet density by comparing them to census data in Mexico. Overall, the Google data had negligible to strong correlation with DENUE data as continuous data and moderate to substantial agreement with DENUE when categorizing. The findings suggest that Google data can be more useful for evaluating food outlet density as a categorical versus a continuous indicator, especially for specific food outlets, such as specialty stores, restaurants, and supermarkets. The results also indicate a better agreement in the least versus most deprived areas.

The evidence on the validity of online geo-referencing services like Google Street View, Google Maps, or Open Street Maps is scarce and mostly related to high-income countries [13, 14]. For example, a study in Spain collected food outlet data from street audits as a gold standard and compared them to Google Maps and Open Street Maps. The results showed a high level of agreement between instruments (measured with Bland–Altman plots) [14]. Another study from Norway conducted a field audit as a gold standard and compared it to Google Street View to assess characteristics of the built environment, such as the number of grocery stores and food outlets. They found moderate to substantial agreement between methods (grocery stores: kappa = 0.56; food outlets: kappa = 0.74) [13]. In our study, we found a substantial agreement across municipalities of 64%. Despite similar results, in contrast with previous studies, we operationalized the food outlets as densities (counts/km^2^). Using a more common characterization of the spatial exposure data, like the density of food outlets (per area or population), can improve the validation or agreement analysis, as this exposure measurement would allow a better assessment of the retail food environment [7]. Moreover, we used two instruments to evaluate the agreement of Google data to identify the density of food outlets as a continuous and categorical variable.

In our study, we found some differences in agreement by type of food outlets. The agreement was higher for restaurants, specialty stores, and supermarkets, and lower for convenience stores and ice cream stores. These results for restaurants and supermarkets are similar to those reported in previous studies. In Norway, the authors found a substantial agreement between virtual and field audits for restaurants (kappa = 0.80) [13]. Another study in Spain showed an almost excellent intraclass correlation for bars/restaurants (ICC = 0.92), fast food restaurants (ICC = 0.86) and supermarkets (ICC = 0.82) [14]. It is hard to explain why some of these differences occur. We believe that one potential explanation is that these venues attract more traffic of people, which may help to keep information up-to-date compared to small retail stores or mini-markets that may be visited only by people living nearby.

Our study also showed better agreement on the least deprived areas, either as indicated for the CCC and Bland Altman plots or weighted kappa that assessed the ability of the Google data to categorize census tracts into equal tertiles of the density of food outlets. In contrast, a systematic review and meta-analysis of studies conducted in high-income countries reported that there were no significant differences in secondary data (i.e., commercially available business) validity across socioeconomic levels [7]. However, some studies described above did not use Google data as a secondary source of information, which may explain why they failed to detect a difference by socioeconomic level. The only study conducted in Latin America is from Brazil and also reported no differences in validity across socioeconomic levels considering sensitivity and positive predictive value [15]. Our study was specifically designed to capture a wide variability of geographic and socioeconomic conditions. Living conditions in Oaxaca de Juarez that are closely linked to Google information, such as access to a cell phone, were very different from those observed in more affluent municipalities. Thus, the differences observed could well reflect these substantial differences introduced by design, a feature that future studies may want to replicate to fully assess the validity of their methods under different living conditions.

The lower agreement measures found in high-deprivation areas may be related to economic development and access to cell phones, especially with an internet connection, since Google Maps collects data from extensive sources, from government agencies to users. Data from the National Survey on Availability and Use of Information Technologies in Households developed by the National Institute of Statistics and Geography of Mexico (INEGI) showed that the proportion of users of smartphones with mobile internet connection was higher in low deprivation areas (92.9%), compared to high deprivation areas (80.9%) [32]. In the municipalities under study, the range of percentage of census tracks with high or very high deprivation was from 10.8% in Hermosillo to 25.5% in Oaxaca de Juarez (see Supplementary Table 1). The different agreement of Google Maps by socioeconomic deprivation could also be related to the concentration of public markets in some areas. While DENUE identified each food outlet inside public markets, Google Maps can only identify a single point of interest. The four municipalities have public markets, but Oaxaca de Juarez also has a central market with hundreds of tenants [33]. Therefore, it is important to keep in mind that Google Maps could perform better in other Latin American countries with the use of cell phones by socioeconomic status similar to Mexico and without an extensive number of public markets.

Our study has some limitations. First, we only included 4 municipalities, therefore, our findings require further assessment before they can be generalized to other contexts. Second, we had limited information on cell phone use and internet access at the municipality level which are key variables that should be considered for future study validations. Third, DENUE does not have information available on non-fixed outlets, like temporary open-air street markets that are open one or two days per week, or street vendors (improvised stalls in public spaces, mobile vendors, or vendors selling from home to home), most of which are informal food establishments; in the case of Google, there is a possibility that it captures some but not all roadside stalls. Therefore, the utility of Google to assess the food environment is limited to formal and informal fixed food establishments. This is particularly important in Latin America where street food vendors have increased over time [34]. In Mexico, 13% of food purchases occur in street markets, street vendors, and acquaintances [35]. Future studies with primary data will be needed to determine the most suitable tool to evaluate the food environment related to non-fixed establishments. Fourth, we do not rule out the possibility that part of the disagreement between DENUE and Google is explained by the different timeframe in which the information from these sources was collected; Google was from April to June 2021, and DENUE refers its data to the entire year of 2021. This is particularly relevant in Mexico since it has a dynamic economy regarding opening and closing establishments. According to the Study on Business Demography conducted by INEGI, from 2020 to 2021 35.5% of establishments closed and 26.6% opened [36]. Finally, due to the granularity of the census tracts, we did not account for errors in georeferencing that could put results in different census tracts.

## Conclusion

In summary, we found that the agreement of Google data as categorical indicator with DENUE data was substantial, suggesting that this instrument could be used to assess the food environment, specifically fixed food outlets. The agreement was better in the least versus most deprived areas. The Google instrument may be used to characterize fixed food outlets in other Latin American countries with similar economic characteristics, given the lack of official and updated data on food retailers in the region, highlighting that in deprived areas the performance of Google data may not be as adequate.

### Supplementary Information

**Supplementary Material 1**.

## Data Availability

Data is available upon request. Please contact the corresponding author/s for further inquiries.
