# Supplementary material for: Agreement between a web collaborative dataset and an administrative dataset to assess the retail food environment in Mexico
Source: BMC Public Health. 2024 Apr 1;24:930. doi: 10.1186/s12889-024-18410-3 (PMC10983718; doi:10.1186/s12889-024-18410-3)
Supplement: Supplementary file 1 — Supplementary Material 1. [file 12889_2024_18410_MOESM1_ESM.docx]

**Supplementary material**

**Supplementary Table 1. Sociodemographic characteristics of the municipalities**

**Supplementary Table 2. Food outlet categories**

**Supplementary Table 3. Search terms for food outlets**

**Supplementary Figure 1. Distribution plot of differences between measurement by Google and DENUE.**

| **Supplementary Table 1. Sociodemographic characteristics of the municipalities** | | | | | |
| --- | --- | --- | --- | --- | --- |
|  | **Total** | **Hermosillo, Sonora** | **Leon, Guanajuato** | **Tlalpan, Mexico City** | **Oaxaca, Oaxaca** |
| **Total census tracts** | **n = 1693** | **n = 662** | **n = 687** | **n = 203** | **n = 141** |
|  |  |  |  |  |  |
| Area (km^2^), [median (p25,p75)] | 0.2 (0.1, 0.4) | 0.2 (0.0, 0.4) | 0.2 (0.1, 0.4) | 0.3 (0.2, 0.5) | 0.3 (0.2, 0.4) |
| Population, [median (p25,p75)] | 1588 (334, 2968) | 857 (116, 2271) | 1745 (474, 3410) | 3015 (1665, 4628) | 1787 (1140, 2462) |
| Socioeconomic deprivation, [n (%)] |  |  |  |  |  |
| Very low | 474 (28.0) | 276 (41.7) | 128 (18.6) | 59 (29.1) | 11 (7.8) |
| Low | 413 (24.4) | 142 (21.5) | 144 (21.0) | 86 (42.4) | 41 (29.1) |
| Medium | 277 (16.4) | 41 (6.2) | 165 (24.0) | 27 (13.3) | 44 (31.2) |
| High | 154 (9.1) | 25 (3.8) | 85 (12.4) | 20 (9.9) | 24 (17.0) |
| Very high | 134 (7.9) | 46 (7.0) | 70 (10.2) | 6 (3.0) | 12 (8.5) |
| Without data | 241 (14.2) | 132 (19.9) | 95 (13.8) | 5 (2.5) | 9 (6.4) |
| p25, 25^th^ percentile; p75, 75^th^ percentile. | | | | | |

| **Supplementary Table 2. Food outlet categories** | | | |
| --- | --- | --- | --- |
| **Food outlet category** | **Food outlet type** | **NAICS codes of the economic unit** | **Examples of food stores** |
| Specialty food stores | Bakery and pastry shops | 461190 - Retail trade of other foods (coffee, bread, bakery, tortillas, eggs) | Traditional stores that sell specialty food (coffee, bread, bakery, tortillas, eggs). These stores could be inside public food markets or not. No chain names available. |
|  | Meat markets | 461121 - Retail trade of red meat + 461122 - Retail trade of poultry + 461123 - Retail trade of fish and seafood + 461150 - Retail trade of milk, other dairy products, and sausages | Traditional stores that sell red meat (raw or semi-cooked beef, pork, lamb, goat and other species of red meat animals), poultry (poultry offal, chicken, quail, duck, turkey), fishmongers (fish or shellfish fresh, dried, salted and frozen, and other marine products), dairy and sausages (milk, cream, butter, yogurt, cheese, chorizo, sausage, mortadella, ham, pork cheese), and other food (coffee, bread, bakery, tortillas, eggs). These stores could be inside public food markets or not. No chain names available. |
|  | Fruit and vegetable stores | 461130 - Retail trade of fresh fruits and vegetables | Traditional stores mainly dedicated to the specialized retail trade of fresh fruits and vegetables. These stores could be inside public food markets or not. No chain names available. |
|  | Health food stores | 464113 - Retail trade of natural products, homeopathic medicines and food supplements | Stores mainly dedicated to the specialized retail trade of natural products (food, herbs and medicinal plants, cosmetics), homeopathic medicines and food supplements for human consumption. |
| Restaurants | Restaurants | 722511 - A la carte or multi-course restaurants + 722512 - Fish and seafood restaurants + 722513 - Snacks (antojitos) restaurants + 722514 - Tacos and sandwiches restaurants + 722518 - Other take-out restaurants | Restaurants mainly dedicated to the preparation of food and beverages (Fish and seafood; snacks, pozole, menudo, birria, quesadillas, gorditas, sopes, barbecue and carnitas; tacos, cakes, sandwiches, hamburgers, and hot dogs) for immediate consumption in the restaurant facilities. These restaurants prepare and serve food and beverages at the same time the customer orders it according to a menu and following specific instructions (à la carte), or they prepare a variety of dishes daily and the customer chooses the one of his preference to be served immediately. Food and beverages are served to the client by staff of the restaurant. |
|  | Bars | 722412 - Drinking places (Alcoholic Beverages) | Venues mainly dedicated to preparing and serving alcoholic beverages for immediate consumption in bars, breweries and pulquerías. |
|  | Coffee shops | 722515 - Cafeterias, soda fountains, ice cream parlours and similar restaurants | Venues mainly dedicated to the preparation of coffee, ice cream, juices, smoothies, and other non-alcoholic beverages, as well as to preparing and serving food for immediate consumption in the facilities and served by the staff of the venue. |
|  | Fast food restaurants | 722516 - Self-service restaurants + 722517 - Pizzas, hamburgers, hot dogs, and roasted chicken take-out restaurants | Restaurants mainly dedicated to the preparation of food and beverages (pizzas, hamburgers, roasted and marinated chickens, hot dogs, and beverages) for immediate consumption in the restaurant facilities. The client orders through a counter and pays before receiving it or serves from a buffet and brings the food and drinks to his place. |
| Candy and ice cream stores | Candy and ice cream stores | 461160 - Retail trade of candies and confectionery raw materials + 461170 - Retail trade of popsicles and ice cream | Local stores that sell candies (sweets, chocolates, chewing gum, bonbons, regional sweets, gelatin powders, flavorings and colorants, and other raw materials used in the manufacture of confectionery products), and ice cream and popsicles. No chain names available. |
| Supermarkets | Supermarkets | 462111 - Retail trade in supermarkets | Bodega Aurrera, Chedraui, Waldos, Soriana, Wal-Mart, Ley, Abarrey, Superama, Comercial Mexicana, H-E-B, Costco, Sams, Mi Bodega, City Market, Tienda ISSSTE, SUPERISSSTE, Tienda IMSS, Mega Comercial Mexicana, Smart, Calimax, City Club, Fresko, BA Express, among others. |
| Convenience stores/small food retail stores | Convenience stores | 462112 - Retail trade in minimarkets | OXXO, 7-Eleven, Circle K, Extra, Bodega Aurrera Express, Super Q, Neto, Pits, Go Mart, Super City, Asturiano, Tiendas 3B, Kiosko, Tent, Chedraui Supercito; and also, local minisupers similar to those franchise. |
|  | Small food retail stores (*abarrotes*) | 461110 - Retail trade in grocery stores | Traditional stores that sell milk, cheese, cream, cold cuts, sweets, cookies, bread, cakes, snacks, fried foods, preserves, canned goods, bottled purified water, soft drinks, juices, and nectars, hydrating drinks, energy drinks, beer, packaged wines and spirits, cigars, egg, toilet paper, detergent, soap, paper napkins, disposable kitchen utensils. No chain names available. |
| Source: Own elaboration adapted from NAICS 2018. The National Institute of Statistics and Geography (INEGI). North American Industry Classification System (NAICS) 2018. Mexico; 2018 | | | |

| **Supplementary Table 3. Search terms for food outlets** | | | | |
| --- | --- | --- | --- | --- |
| **Food outlet category** | **NAICS codes of the economic unit** | | **Record** | **Search terms** |
| Specialty food stores | 461190 | Retail trade of other foods (coffee, bread, bakery, tortillas, eggs) | 1 | Botana |
|  |  |  | 2 | Churreria |
|  |  |  | 3 | Dureria |
|  |  |  | 4 | Elotes y esquites |
|  |  |  | 5 | Expendio de huevo |
|  |  |  | 6 | Expendio de pan |
|  |  |  | 7 | Frituras |
|  |  |  | 8 | Panaderia |
|  |  |  | 9 | Panificadora |
|  |  |  | 10 | Pasteleria |
|  |  |  | 11 | Tamaleria |
|  |  |  | 12 | Tortilleria |
|  |  |  | 13 | Tostaduria |
|  | 461121 | Retail trade of red meat | 14 | Bodega de carne |
|  |  |  | 15 | Carniceria |
|  |  |  | 16 | Congelada |
|  |  |  | 17 | Expendio de carne |
|  |  |  | 18 | Expendio de viscera |
|  |  |  | 19 | TSK |
|  | 461122 | Retail trade of poultry | 20 | Bodega de polleria |
|  |  |  | 21 | Expendio de pollo |
|  |  |  | 22 | Polleria |
|  |  |  | 23 | Pollo fresco |
|  | 461123 | Retail trade of fish and seafood | 24 | Pescados y mariscos |
|  |  |  | 25 | Bahia Kino |
|  | 461150 | Retail trade of milk, other dairy products and sausages | 26 | Carnes frias |
|  |  |  | 27 | Cremeria |
|  |  |  | 28 | Expendio de leche |
|  |  |  | 29 | Lecheria |
|  |  |  | 30 | Queseria |
|  |  |  | 31 | Salchichoneria |
|  | 461130 | Retail trade of fresh fruits and vegetables | 32 | Bodega de fruta |
|  |  |  | 33 | Chiles |
|  |  |  | 34 | Frutas y legumbres |
|  |  |  | 35 | Frutas y verduras |
|  |  |  | 36 | Fruteria |
|  |  |  | 37 | Recauderia |
|  |  |  | 38 | Verduleria |
|  | 464113 | Retail trade of natural products, homeopathic medicines, and food supplements | 39 | Casa naturista |
|  |  |  | 40 | Farmacia naturista |
|  |  |  | 41 | GNC |
|  |  |  | 42 | Herbalife |
|  |  |  | 43 | Productos naturistas |
|  |  |  | 44 | Tienda naturista |
|  |  |  | 45 | Tiendas de alimentos para deportistas y suplementos |
|  |  |  | 46 | Tiendas de suplemento |
|  |  |  | 47 | Tiendas de vitamina |
| Restaurants | 722511 | A la carte or multi-course restaurants | 48 | Almuerzo |
|  |  |  | 49 | Chilis |
|  |  |  | 50 | Comedor |
|  |  |  | 51 | Comida china |
|  |  |  | 52 | Comida corrida |
|  |  |  | 53 | Merendero |
|  |  |  | 54 | Parrillada |
|  |  |  | 55 | Restaurant |
|  | 722512 | Fish and seafood restaurants | 56 | Cocteleria |
|  |  |  | 57 | Marisco |
|  |  |  | 58 | Marisqueria |
|  |  |  | 59 | Pescaderia |
|  | 722513 | Snacks (antojitos) restaurants | 60 | Antojeria |
|  |  |  | 61 | Antojito |
|  |  |  | 62 | Barbacoa |
|  |  |  | 63 | Birria |
|  |  |  | 64 | Birrieria |
|  |  |  | 65 | Carnita |
|  |  |  | 66 | Cena |
|  |  |  | 67 | Cenaduria |
|  |  |  | 68 | Empanada |
|  |  |  | 69 | Ensalada |
|  |  |  | 70 | Fonda |
|  |  |  | 71 | Gorditas |
|  |  |  | 72 | Huaraches |
|  |  |  | 73 | Loncheria |
|  |  |  | 74 | Menuderia |
|  |  |  | 75 | Pozole |
|  |  |  | 76 | Pozoleria |
|  |  |  | 77 | Quesadilla |
|  |  |  | 78 | Sopes |
|  |  |  | 79 | Taqueria |
|  | 722514 | Tacos and sandwiches restaurants | 80 | Burrito |
|  |  |  | 81 | Guacamaya |
|  |  |  | 82 | Puesto de taco |
|  |  |  | 83 | Puesto de torta |
|  |  |  | 84 | Tacos |
|  |  |  | 85 | Torta |
|  |  |  | 86 | Torteria |
|  |  |  | 87 | Venta de comida |
|  | 722518 | Other take-out restaurants | 88 | Cocina economica |
|  |  |  | 89 | Comida casera |
|  |  |  | 90 | Comida economica |
|  |  |  | 91 | Comida para llevar |
|  | 722412 | Drinking places (Alcoholic Beverages) | 92 | Bar |
|  |  |  | 93 | Cantina |
|  |  |  | 94 | Centro Botanero |
|  |  |  | 95 | Cerveceria |
|  |  |  | 96 | Cheleria |
|  |  |  | 97 | Mezcaleria |
|  |  |  | 98 | Pub |
|  |  |  | 99 | Pulqueria |
|  | 722515 | Cafeterias, soda fountains, ice cream parlours and similar restaurants | 100 | Aguas frescas |
|  |  |  | 101 | Almuersos |
|  |  |  | 102 | Cafes |
|  |  |  | 103 | Cafeteria |
|  |  |  | 104 | Chocomile |
|  |  |  | 105 | Dairy Queen |
|  |  |  | 106 | Desayuno |
|  |  |  | 107 | Fuente de Soda |
|  |  |  | 108 | Jugo |
|  |  |  | 109 | Jugueria |
|  |  |  | 110 | Licuado |
|  |  |  | 111 | Neveria |
|  |  |  | 112 | Paleteria |
|  |  |  | 113 | Refresqueria |
|  |  |  | 114 | Tienda escolar |
|  |  |  | 115 | Tiendita de la escuela |
|  |  |  | 116 | Tiendita escolar |
|  | 722516 | Self-service restaurants | 117 | Carls J |
|  |  |  | 118 | Comida rapida |
|  |  |  | 119 | Domino’s Pizza |
|  |  |  | 120 | Fast-food |
|  |  |  | 121 | Kentucky Fried Chicken |
|  |  |  | 122 | KFC |
|  |  |  | 123 | Mc Donalds |
|  |  |  | 124 | Pizza Hut |
|  |  |  | 125 | Subway |
|  | 722517 | Pizzas, hamburgers, hot dogs, and roasted chicken take-out restaurants | 126 | Hamburguesa |
|  |  |  | 127 | Hamburguesas al carbon |
|  |  |  | 128 | Hot dogs |
|  |  |  | 129 | Pizza |
|  |  |  | 130 | Pizzeria |
|  |  |  | 131 | Polleria |
|  |  |  | 132 | Pollos asados |
|  |  |  | 133 | Pollos rostizados |
|  |  |  | 134 | Rosticeria |
| Candy and ice cream stores | 461160 | Retail trade of candies and confectionery raw materials | 135 | Chocolateria |
|  |  |  | 136 | Dulceria y materias primas |
|  |  |  | 137 | Dulces |
|  |  |  | 138 | Puesto de dulce |
|  |  |  | 139 | Tienda de dulce |
|  | 461170 | Retail trade of popsicles and ice cream | 140 | Heladeria |
|  |  |  | 141 | Helados |
|  |  |  | 142 | Nieves |
| Supermarkets | 462111 | Retail trade in supermarkets | 143 | Abarrey |
|  |  |  | 144 | BA Express |
|  |  |  | 145 | Bodega Aurrera |
|  |  |  | 146 | Calimax |
|  |  |  | 147 | Chedraui |
|  |  |  | 148 | City Clubresko |
|  |  |  | 149 | City Market |
|  |  |  | 150 | Comercial Mexicana |
|  |  |  | 151 | Costco |
|  |  |  | 152 | HEB |
|  |  |  | 153 | Hipermercado |
|  |  |  | 154 | Ley |
|  |  |  | 155 | Mi Bodega |
|  |  |  | 156 | Sams |
|  |  |  | 157 | Smart |
|  |  |  | 158 | Soriana |
|  |  |  | 159 | Superama |
|  |  |  | 160 | SUPERISSSTE |
|  |  |  | 161 | supermarket |
|  |  |  | 162 | Supermercado |
|  |  |  | 163 | Tienda IMSS |
|  |  |  | 164 | Tienda ISSSTE |
|  |  |  | 165 | Wal Mart |
|  |  |  | 166 | Waldos |
| Convenience stores/small food retail stores | 462112 | Retail trade in minimarkets | 167 | Mini super |
|  |  |  | 168 | Oxxo |
|  |  |  | 169 | Tienda de conveniencia |
|  | 461110 | Retail trade in grocery stores | 170 | Abarrote |
|  |  |  | 171 | Miscelanea |
|  |  |  | 172 | Tendajon |
|  |  |  | 173 | Tienda de abarrote |
|  |  |  | 174 | Tienda de alimentos |
|  |  |  | 175 | Tienda de comestible |
|  |  |  | 176 | Tienda de ultramarino |
|  |  |  | 177 | Tiendita de abarrote |

**Supplementary Figure 1. Distribution plot of differences between measurement by Google and DENUE.**
